# Appetite Perceptions, Gastrointestinal Symptoms, Ghrelin, Peptide YY and State Anxiety Are Disturbed in Adolescent Females with Anorexia Nervosa and Only Partially Restored with Short-Term Refeeding

**DOI:** 10.3390/nu11010059

**Published:** 2018-12-28

**Authors:** Gabriella A. Heruc, Tanya J. Little, Michael Kohn, Sloane Madden, Simon Clarke, Michael Horowitz, Christine Feinle-Bisset

**Affiliations:** 1 Adelaide Medical School and National Health and Medical Research Council of Australia Centre of Research Excellence in Translating Nutritional Science to Good Health, Level 5 Adelaide Health and Medical Sciences Building, Corner North Terrace and George Street, Adelaide 5005, Australia; gabriella.heruc@health.nsw.gov.au (G.A.H.); tanya.little@adelaide.edu.au (T.J.L.); michael.horowitz@adelaide.edu.au (M.H.); 2 The Children’s Hospital at Westmead, Sydney 2145, Australia; michael.kohn@health.nsw.gov.au (M.K.); sloane.madden@health.nsw.gov.au (S.M.); 3 Adolescent and Young Adult Medicine Department, Westmead Hospital, Sydney 2145, Australia; simon.clarke@sydney.edu.au

**Keywords:** bloating, cholecystokinin, fullness, gastrointestinal hormones, hunger, pancreatic polypeptide, malnutrition, nutritional rehabilitation, starvation, stress

## Abstract

Factors underlying disturbed appetite perception in anorexia nervosa (AN) are poorly characterized. We examined in patients with AN whether fasting and postprandial appetite perceptions, gastrointestinal (GI) hormones, GI symptoms and state anxiety (i) differed from healthy controls (HCs) and (ii) were modified by two weeks of refeeding. 22 female adolescent inpatients with restricting AN, studied on hospital admission once medically stable (Wk0), and after one (Wk1) and two (Wk2) weeks of high-calorie refeeding, were compared with 17 age-matched HCs. After a 4 h fast, appetite perceptions, GI symptoms, state anxiety, and plasma acyl-ghrelin, cholecystokinin (CCK), peptide tyrosine tyrosine (PYY) and pancreatic polypeptide (PP) concentrations were assessed at baseline and in response to a mixed-nutrient test-meal (479 kcal). Compared with HCs, in patients with AN at Wk0, baseline ghrelin, PYY, fullness, bloating and anxiety were higher, and hunger less, and in response to the meal, ghrelin, bloating and anxiety were greater, and hunger less (all *p* < 0.05). After two weeks of refeeding, there was no change in baseline or postprandial ghrelin or bloating, or postprandial anxiety, but baseline PYY, fullness and anxiety decreased, and baseline and postprandial hunger increased (*p* < 0.05). We conclude that in AN, refeeding for 2 weeks was associated with improvements in PYY, appetite and baseline anxiety, while increased ghrelin, bloating and postprandial anxiety persisted.

## 1. Introduction

Anorexia nervosa (AN) is characterised by severe dietary restriction, weight loss and high levels of anxiety [1,2]. Gastrointestinal (GI) symptoms (e.g., bloating and nausea) are commonly reported [3] and may contribute to a reduction in caloric intake. Moreover, despite significant malnutrition [4,5], patients frequently report reduced hunger and increased fullness [6,7]. It is, however, unclear whether these self-reported symptoms are related to alterations in the GI mechanisms underlying appetite regulation or to heightened anxiety.

In healthy individuals, the GI tract plays a pivotal role in appetite regulation. Slowed gastric emptying, and in particular, increased content of the distal stomach (antral distension), are related directly to greater fullness [8]. Moreover, as nutrients reach the small intestine, GI hormones (e.g., cholecystokinin (CCK) [9], peptide tyrosine tyrosine (PYY) [10], pancreatic polypeptide (PP) [11]) are released, and ghrelin is suppressed [12], providing feedback to further slow gastric emptying and reduce food intake. Both gastric emptying and GI hormone secretion are sensitive to changes in diet [13,14]. For example, a 4-day fast slows gastric emptying in healthy individuals [13], and 30% dietary restriction for 12 weeks modifies postprandial GI hormone release in obesity [14]. Thus, in AN, prolonged energy restriction has the potential to induce changes in the GI mechanisms involved in appetite regulation. Previous research has reported that both fasting and postprandial total ghrelin and PYY(3-36) concentrations are higher in patients with AN than healthy individuals, with levels decreasing towards those in healthy individuals after three months of refeeding [15]. Observations in relation to CCK and PP in untreated patients with AN have been inconsistent, with plasma concentrations increased [16,17], or not different [18,19], from healthy controls (HCs). Gastric emptying is also severely delayed in AN [19,20,21], and we have reported recently, in the current cohort, that two weeks of standardized high-calorie refeeding improves gastric emptying [21]. However, it remains unclear whether changes in GI hormones contribute to the disturbed appetite perceptions reported by patients with AN and to what extent these factors may improve with short-term refeeding.

Anxiety, a primary feature of AN, can potently influence appetite in healthy individuals, to potentially increase or decrease hunger and food intake [22,23]. Psychological stress also appears to influence GI motility [24,25] and GI hormone secretion [26]. Moreover, GI symptoms (e.g., nausea, bloating and epigastric discomfort) are frequent complaints in patients with AN [27]. In other conditions, such as functional dyspepsia, which is also associated with high levels of anxiety [28], there is a positive association between anxiety levels with postprandial symptom intensity [29]. Interestingly, functional dyspepsia is also associated with disturbances in GI motility [30] and circulating ghrelin [31], CCK and PYY [32] concentrations. It is, therefore, conceivable that anxiety may contribute to the appetite and GI disturbances, and GI symptoms, in AN. In AN, greater pre-meal anxiety is associated with reduced food intake [33], and pre- and post-meal anxiety is at least partially reduced after nutritional rehabilitation [34].

We, therefore, investigated in malnourished patients with AN whether appetite perceptions, GI hormones, GI symptoms and state anxiety at baseline, and in response to a mixed-nutrient semi-solid test-meal (i) differed from HCs and (ii) were modified by short-term refeeding.

## 2. Materials and Methods

### 2.1. Participants

Twenty-two female adolescent inpatients with AN (restricting sub-type), as defined by the Diagnostic and Statistical Manual of Mental Disorders [35]) criteria (age: 15.9 ± 0.4 years, range 13–19 years) and 17 healthy, age-matched female control participants (16.5 ± 0.7 years, range 12–19 years) were included in the study. Fifteen of the 22 patients had not received previous treatment and reported 36 ± 4 weeks of recent dietary restriction (weight loss: 12 ± 1 kg; total illness duration: 11 ± 3 months) [21]. The 7 patients who had been previously treated reported 10 ± 2 weeks of recent dietary restriction (weight loss: 6 ± 1 kg; total illness duration: 27 ± 6 months). Based on previous studies [36,37,38], sample sizes of *n* = 22 patients with AN and *n* = 17 HCs were powered to detect a difference between groups in fasting PYY of 42.5 pg/mL, and a difference in postprandial PYY at 120 min of 31.6 pg/mL, with *p* < 0.05 and statistical power (1-β) 80%. These numbers also allowed detection of a mean difference of 24 mm on 100-mm visual analogue scales (VAS) for hunger and fullness, based on post-hoc power calculations of previous research [6].

Participant recruitment and characteristics have been described previously [21]. Patients were excluded if they had current diarrhoea, constipation, GI disease or previous surgery, or a history of other medical conditions unrelated to complications of AN-related malnutrition, vomiting, smoking or consumption of >20 g alcohol per day. One patient took olanzapine throughout the study and another patient during week 1 only, but there was no use of any other medication.

The study protocol was approved by the Human Research Ethics Committees at the Sydney Children’s Hospital Network (reference no. 11CHW88, approval: 23 May 2011), and ratified by the Royal Adelaide Hospital and the University of Adelaide, and all studies were carried out in accordance with the Declaration of Helsinki. All participants, and parents for those <18 years old, provided informed, written consent prior to their enrolment. The study was registered as a clinical trial with the Australia and New Zealand Clinical Trial Registry (http://www.anzctr.org.au; Trial ID: 12616000134426).

### 2.2. Study Design

Upon admission, all patients with AN commenced a standardized rapid high-calorie refeeding protocol commencing on 2400 kcal/day (100 mL/h; Jevity, Abbott Nutrition (1 kcal/mL)), and then transitioned to an energy delivery of 2400–3200 kcal/day [39].

Patients were studied on three occasions: once medically stable within the first five days (2.2 ± 0.2 days) of admission (Wk0), then one (Wk1) and two (Wk2) weeks post-Wk0 while undergoing refeeding treatment. HCs were studied on one occasion only. On each study day, appetite perceptions, plasma acyl-ghrelin, CCK, PYY and PP concentrations, as well as GI symptoms and state anxiety were evaluated at baseline and in response to an oral mixed-nutrient test-meal.

### 2.3. Protocol

Patients were studied during their inpatient admission and, if discharged prior to Wk2 (*n* = 6), re-attended hospital. Both discharged patients and HCs were asked to refrain from vigorous exercise and alcohol intake for 24 h before the study, and on the study day, attended the hospital at 8:00 a.m. after fasting from 8:00 p.m. the night before.

On each study day, participants were weighed in the morning. To allow for regular blood sampling, an intravenous cannula was inserted into an antecubital vein at 8:00 a.m. At 8:30 a.m., patients with AN on an oral diet (*n* = 17 at Wk0, *n* = 22 at Wk1 and Wk2) and all HCs were provided with a standardised breakfast (30 g cereal wheat biscuits (Weetbix, Sanitarium, Australia), 400 mL full-cream milk and an apple; 479 kcal, 65 g carbohydrate, 19 g protein, 15 g fat, 7 g fibre), and allowed up to 30 min for consumption. Medically stable patients still receiving continuous nasogastric feeds (100 mL/h; Jevity, Abbott Nutrition (1 kcal/mL)) at Wk0 (*n* = 5) had feeds ceased at 9:00 a.m. in lieu of the standardised breakfast. No further food or fluid (except water) was consumed prior to the test-meal.

At 1:00 p.m., before consumption of the test-meal (*t* = 0 min), a baseline blood sample (15 mL) was collected, and, using VAS questionnaires, participants rated appetite perceptions, GI symptoms (nausea and bloating) and state anxiety (baseline values). Following this, a mixed-nutrient semi-solid test-meal identical to that given at breakfast was consumed over 15 min. Further blood samples and VAS ratings were collected at *t* = 30, 60 and 120 min, with timing commencing as soon as test-meal ingestion was complete. At the end of the study day, participants were provided with afternoon tea before returning to the ward or home.

### 2.4. Measurements

#### 2.4.1. Appetite Perceptions, GI Symptoms and State Anxiety

Appetite (hunger, desire to eat, prospective consumption and fullness), nausea, bloating and state anxiety were assessed with a previously described 100-mm VAS questionnaire, anchored by ‘not at all’ and ‘very much’ [40]. Assessment of state anxiety by VAS has been validated in adolescents against the State-Trait Anxiety Inventory (STAI) [41]. A broad range of GI symptoms (including nausea, sickness, vomiting, bloating, abdominal cramps, early satiety, acidic eructation/heartburn, loss of appetite, retrosternal discomfort and epigastric pain/upper abdominal pain) were assessed using the validated GI Symptom Score (GIS) questionnaire [42] once on each study day at baseline.

#### 2.4.2. Plasma Hormone Analysis

Blood samples were collected in ice-chilled EDTA-treated tubes, containing 20 uL/mL blood of the serine protease inhibitor, 4-(2-aminoethyl) benzene sulfonyl fluoride hydrochloride (Pefabloc, Roche, Australia) [43]. Samples were separated by centrifugation (3200 rpm, 15 min, 4 °C) within 15 min of collection, and plasma stored at −80 °C until assayed, for later analysis of plasma concentrations of acyl-ghrelin, CCK-8, PYY and PP. Plasma acyl-ghrelin (pg/mL), total PYY (pg/mL) and PP (pg/mL) concentrations were measured using a multiplex assay (Milliplex^®^ MAP Human Metabolic Hormone Magnetic Bead Panel, HMHEMAG-34K, Millipore Corporation, Temecula, CA, USA) and analysed on a Bio-plex^®^ MAGPIXTM Multiplex Reader (Luminex^®^, Millipore Corporation, Temecula, CA, USA) using xPONENT^®^ software (Luminex^®^, Millipore Corporation, Temecula, CA, USA, version 4.2) according to manufacturer’s instructions. There was negligible antibody cross-reactivity. The minimum detectable limits were 13 pg/mL for ghrelin, 28 pg/mL for PYY and 2 pg/mL for PP. Intra- and inter-assay coefficients of variation (CVs) were <10% and <15%, respectively, for all analytes. Plasma CCK-8 (pmol/L) was measured by radioimmunoassay using an adaption of the method of Santangelo et al. [44]. Samples were extracted in 66% ethanol, extracts were dried down and resuspended in assay buffer (50 mM phosphate, 10 mM EDTA, 2 g/L gelatin, pH 7.4). Standards were prepared using synthetic sulphated CCK-8 (Sigma Chemical, St Louis, MO, USA), antibody (C2581, Lot 105H4852, Sigma Chemical, St Louis, MO, USA) was added at a working dilution of 1/17,500 and sulphated CCK-8 125I-labeled with Bolton and Hunter reagent (Perkin Elmer, Boston, MA, USA) was used as tracer. Incubation was for 7 days at 4 °C. The antibody bound fraction was separated by the addition of dextran-coated charcoal containing gelatin (0.015 g gelatin, 0.09 g dextran, 0.15 g charcoal in 30 mL assay buffer) and the radioactivity determined in the supernatants following centrifugation. Intra- and inter-assay CVs were 6.2% and 13.4%, respectively. The minimal detectable limit was 1 pmol/L.

### 2.5. Data and Statistical Analyses

The analyses were performed in collaboration with a professional biostatistician.

Differences in EDE-Q (Eating Disorder Examination Questionnaire) and RCADS (Revised Children’s Anxiety and Depression Scale) between patients with AN and HCs were analysed using independent samples t-tests. Body weight was expressed as percentage of expected body weight (%EBW), determined using the 50th percentile for body mass index (BMI; kg/m^2^) for age and sex from Centers for Disease Control and Prevention (USA) growth charts (37).

Baseline and responses to the test meal. Incremental areas under the curves (iAUCs) were calculated using the trapezoidal rule (correcting for differences in baseline (pre-meal) concentrations to represent the magnitude of the response to the test meal) for all variables. Where data decreased from baseline (i.e., ghrelin, hunger, desire to eat and prospective consumption), inverse iAUCs were calculated. For gut hormones, iAUC values were divided by the time of last measurement to obtain a final weighted average to account for plasma samples that could not be collected in *n* = 3 participants (on one occasion each) due to cannula failure at *t* = 120 min. For consistency, iAUCs for all other variables were also divided by 120 to obtain a final weighted average.

Longitudinal comparisons. GI hormone data were normally distributed and analysed using repeated-measures ANOVAs with visit (Wk0, Wk1, Wk2) as the factor. Post-hoc paired comparisons, adjusted for multiple comparisons by Bonferroni’s correction, were performed when ANOVAs revealed significant effects. To compare patients with AN (at both Wk0 and Wk2) with HCs, baseline values and iAUCs were analysed using independent samples t-tests. Appetite, bloating, nausea and anxiety VAS scores were skewed and analysed using the Friedman test. Non-parametric Mann-Whitney tests were used to compare baseline values and iAUCs for GI symptoms and anxiety scores in patients with AN (at Wk0 and Wk2) with HCs.

Data were analysed using IBM SPSS Statistics for Windows, version 23.0 (IBM Corp., released 2015, Armonk, NY, USA). Statistical significance was accepted at *p* < 0.05. Parametric data are presented as means ± standard errors (SEs), and non-parametric data as medians (25th–75th quartiles).

## 3. Results

The experimental conditions were well tolerated by all study participants. At Wk0, %EBW was less in patients with AN than in HCs (78.7% ± 1.8 vs. 102.8% ± 2.2, *p* < 0.001). In patients, %EBW was higher at Wk1 (83.8% ± 1.8) and Wk2 (85.2% ± 1.9) compared with Wk0 (both *p* < 0.001), and higher at Wk2 compared with Wk1 (*p* < 0.05) [21]. At Wk2, %EBW remained lower in patients than in HCs (*p* < 0.001).

Patients with AN had significantly higher scores on both the EDE-Q global score [45] (3.1 ± 0.3 vs. 0.5 ± 0.1, *p* < 0.05) and RCADS Total Anxiety score [46] (55.9 ± 2.6 vs. 39.6 ± 1.5, *p* < 0.05).

### 3.1. Appetite Perceptions

**Figure 1:**
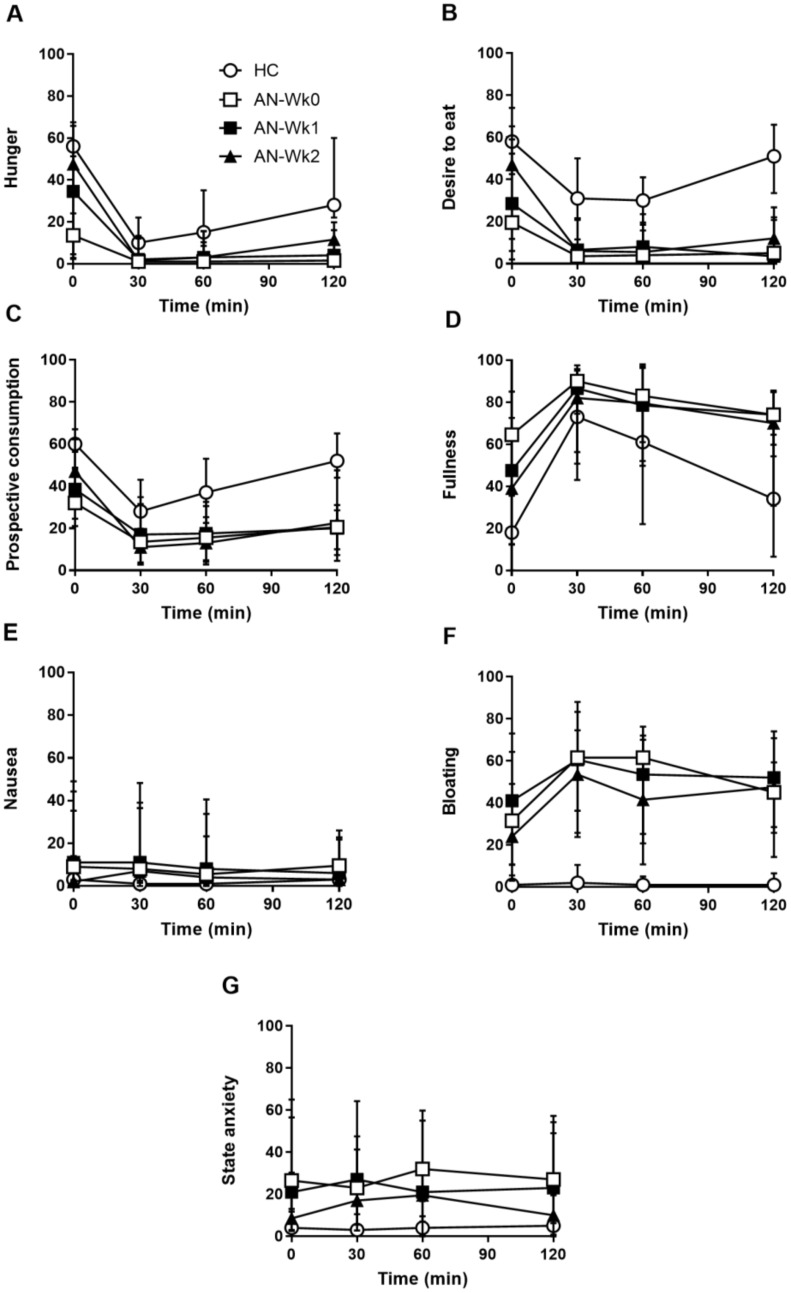
Hunger (**A**), desire to eat (**B**), prospective consumption (**C**), fullness (**D**) nausea (**E**), bloating (**F**) and state anxiety (**G**) scores before and after a mixed-nutrient, solid-liquid meal in 22 adolescent females with anorexia nervosa on admission (AN-Wk0), and after one (AN-Wk1) and two (AN-Wk2) weeks of refeeding, as well as in 17 age-matched healthy controls (HCs). Data are medians (25th–75th quartiles).

Baseline ratings (t = 0 min). At Wk0, baseline hunger, desire-to-eat and prospective consumption were less, and fullness greater, in patients than HCs (*p* < 0.05) (Table 1, Figure 1A–D). In patients, baseline hunger, desire-to-eat, prospective consumption and fullness did not differ between Wk0, Wk1 and Wk2, although median values for hunger, desire-to-eat and prospective consumption increased, and for fullness decreased. At Wk2, there were no longer any differences in baseline hunger, desire-to-eat or prospective consumption between patients and HCs, but there was a trend for baseline fullness to remain higher in patients than in HCs (*p* = 0.085).

Responses to the test meal. At Wk0, hunger inverse iAUC was less in patients than HCs (*p* < 0.05) (Table 2). However, there was no difference in fullness iAUC, or desire-to-eat and prospective consumption inverse iAUCs, between patients and HCs. In patients, fullness iAUC and hunger, desire-to-eat and prospective consumption inverse iAUCs did not differ between Wk0, Wk1 and Wk2. At Wk 2, there were no differences in fullness iAUC, or hunger, desire-to-eat or prospective consumption inverse iAUCs, between patients and HCs.

### 3.2. Gastrointestinal Symptoms

Baseline ratings. At Wk0, the total GIS score was greater in patients than HCs (*p* < 0.05) (Table 1). In patients, the GIS was lower at Wk1 and Wk2 than Wk0 (*p* < 0.05), but there was no difference between Wk1 and Wk2. At Wk2, the GIS remained greater in patients than in HCs (*p* < 0.05).

At Wk0, there was no difference in nausea between patients and HCs, but bloating was greater in patients (*p* < 0.05) (Table 1, Figure 1E,F). In patients, nausea and bloating did not differ between Wk0, Wk1 and Wk2. At Wk2, bloating, but not nausea, was greater in patients than HCs (*p* < 0.05).

Responses to the test meal. At Wk0, bloating, but not nausea, iAUC was greater in patients than HCs (*p* < 0.05) (Table 2, Figure 1E,F). In patients, nausea and bloating iAUC did not differ between Wk0, Wk1 and Wk2. At Wk2, bloating, but not nausea, iAUC was greater in patients than in HCs (*p* < 0.05).

### 3.3. State Anxiety

Baseline ratings. At Wk0, baseline anxiety was higher in patients than HCs (*p* < 0.05) (Table 1, Figure 1G). In patients, there was a trend for a treatment effect for baseline anxiety (*p* = 0.06), with mean values declining over time, but there was no difference between Wk0, Wk1 and Wk2. At Wk2, there was also no difference in baseline anxiety between patients and HCs.

Responses to the test meal. At Wk0, anxiety iAUC was greater in patients with AN than in HCs (*p* < 0.05) (Table 2, Figure 1G). In patients, anxiety iAUC did not differ between Wk0, Wk1 and Wk2. At Wk2, anxiety iAUC was greater in patients than in HCs (*p* < 0.05).

### 3.4. Gut Hormones

#### 3.4.1. Plasma Acyl-Ghrelin

**Figure 2:**
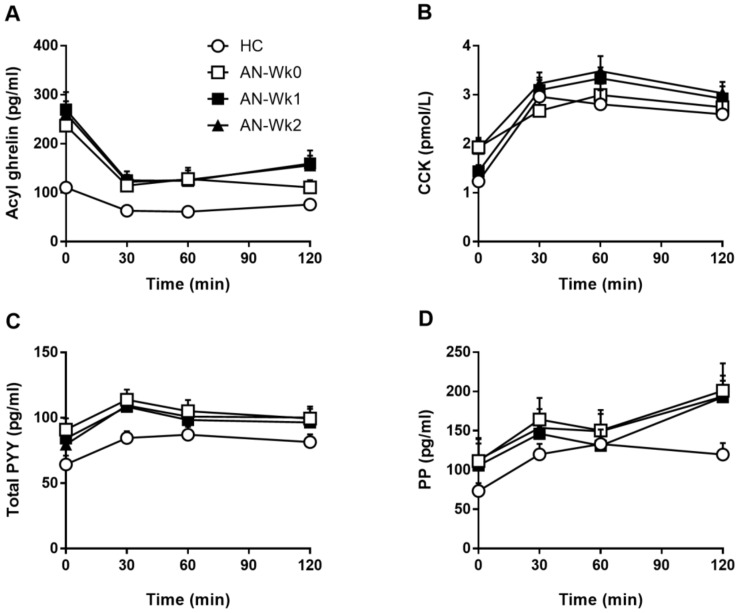
Acyl ghrelin (**A**), CCK (**B**) total PYY (**C**), and PP (**D**) concentrations before and after a mixed-nutrient, solid-liquid meal in 22 adolescent females with anorexia nervosa on admission (AN-Wk0), and after one (AN-Wk1) and two weeks (AN-Wk2) of refeeding, as well as in 17 age-matched healthy controls (HCs). Data are means ± SEM. For CCK: *n* = 16 HCs and *n* = 19 patients with AN due to missing data.

Baseline concentrations. At Wk0, baseline ghrelin was higher in patients than HCs (*p* < 0.05) (Table 3, Figure 2A). In patients, baseline ghrelin did not differ between Wk0, Wk1 and Wk2, and, at Wk2, baseline ghrelin remained higher than in HCs (*p* < 0.05).

Responses to the test meal. At Wk0, ghrelin inverse iAUC was greater in patients with AN than in HCs (*p* < 0.05) (Table 4, Figure 2A). In patients, ghrelin inverse iAUC did not differ between Wk0, Wk1 and Wk2, and, at Wk2, ghrelin inverse iAUC remained greater than in HCs (*p* < 0.05).

#### 3.4.2. Plasma CCK

Baseline concentrations. There was no difference in baseline CCK between patients and HCs at Wk0 or Wk2, nor in patients between Wk0, Wk1 and Wk2 (Table 3, Figure 2B).

Responses to the test meal. There were no differences in CCK iAUC between patients with AN and HCs at Wk0 or Wk2, nor in patients across Wk0, Wk1 and Wk2 (Table 4, Figure 2B).

#### 3.4.3. Plasma Total PYY

Baseline concentrations. At Wk0, baseline PYY concentrations were higher in patients than HCs (*p* < 0.05) (Table 3, Figure 2C). In patients, baseline PYY did not differ between Wk0, Wk1 and Wk2. At Wk2, there was no difference in baseline PYY between patients and HCs.

Responses to the test meal. There were no differences in PYY iAUC between patients with AN and HCs at Wk0 or Wk2, nor in patients across Wk0, Wk1 and Wk2 (Table 4, Figure 2C).

#### 3.4.4. Plasma PP

Baseline concentrations. There was no difference in baseline PP concentrations between patients and HCs at Wk0 or Wk2, nor in patients between Wk0, Wk1 and Wk2 (Table 3, Figure 2D).

Responses to the test meal. There were no differences in PP iAUC between patients with AN and HCs at Wk0 or Wk2, nor in patients across Wk0, Wk1 and Wk2 (Table 4, Figure 2D).

## 4. Discussion

The current study established that malnourished patients with AN display greater fullness, bloating and overall GI symptoms, and less hunger, desire to eat and prospective consumption when compared with HCs, and that two weeks of high-calorie refeeding was associated with changes in ratings of hunger, prospective consumption, desire to eat and fullness towards normal, while bloating and GIS score remained higher. The patients also exhibited disturbances in GI hormones. At Wk0, baseline concentrations of acyl-ghrelin and total PYY were higher, while postprandial suppression of ghrelin was greater than in HCs; at Wk2, the disturbances in ghrelin remained, but PYY no longer differed from HCs. At Wk0, baseline and postprandial anxiety were also greater in patients than in HCs, and while baseline anxiety improved with refeeding, postprandial anxiety remained higher in patients than in HCs at Wk2.

Patients with AN exhibited substantially lower fasting and postprandial hunger and greater fullness than HCs, despite chronically restricted energy intake prior to Wk0. These observations are consistent with previous studies [6,7]. It has been suggested that patients with AN may be unaware of, or not respond to, internal and external eating-related cues in a manner comparable to healthy people [47]. Refeeding was associated with increases in baseline hunger, desire to eat and prospective consumption, as well as the suppression of hunger in response to the test-meal, suggesting that refeeding partially restores perceptions of appetite. Reduced hunger and increased fullness evident in the patients may reflect pathophysiologically enhanced sensitivity to the appetite-suppressant effect of nutrients, which appears to be, at least in part, reversed by re-exposure to feeding. Indeed, sensitivity to nutrients is altered in other conditions, including obesity [48], functional dyspepsia [49] and anorexia of ageing [50]. Moreover, in obesity, the altered sensitivity can be modified by changes in dietary intake [14,51]. For example, while intraduodenal lipid infusion failed to suppress hunger in obese individuals at baseline, following 4 days of 70% dietary energy restriction lipid potently suppressed hunger, and this was associated with a reduction in *ad libitum* energy intake [51]. Of note, these changes in appetite and food intake were associated with greater lipid-induced suppression of ghrelin and stimulation of PYY [51], demonstrating that intestinal nutrient sensing mechanisms underlie, at least in part, the adaptation of appetite regulation following changes in nutritional status.

We also found disturbances in fasting and postprandial concentrations of gut hormones in AN. Ghrelin stimulates hunger and food intake in healthy individuals [52,53]. Patients had elevated acyl-ghrelin at baseline, and a greater suppression of ghrelin following the test meal when compared with HCs, and these responses did not change following two weeks of refeeding, consistent with previous studies [54,55,56,57,58]. It has been suggested that elevated acyl-ghrelin is an adaptive response to increase hunger and energy intake following chronic nutrient restriction in AN. Despite this, at Wk0, baseline and postprandial hunger were markedly less, suggesting a disconnect between acyl-ghrelin and hunger in AN. Moreover, after two weeks of refeeding, patients reported greater hunger, while ghrelin remained unchanged, suggesting that with increased nutrient exposure, patients with AN became more sensitive to the orexigenic actions of acyl-ghrelin. Previous studies have also reported that circulating levels of desacyl-ghrelin, a degradation product of acyl-ghrelin, which has been shown in animal studies to suppress food intake [59] and to inhibit the orexigenic effect of acyl-ghrelin [60], are substantially higher in patients with AN than in controls [54,61]. Moreover, in a small study of patients with AN, desacyl-ghrelin concentrations were shown to decrease after just one week of refeeding [61], thus, this may be another potential mechanism by which hunger perceptions are restored following acute refeeding. We did not measure desacyl-ghrelin concentrations.

At Wk0, baseline, but not postprandial, PYY concentrations were elevated in patients with AN, consistent with previous observations [15,37,62], while after two weeks of refeeding, baseline PYY no longer differed between patients and HCs, consistent with findings of reduced PYY(3-36) after ~3 months of nutritional rehabilitation [15] and reduced total PYY after weight restoration [62]. PYY is an anorexigenic gut peptide [63], and elevated baseline levels of PYY could mediate the reduction in hunger ratings and increased fullness reported by patients at Wk0, while the decrease in baseline PYY at Wk2 may contribute to the improvements in appetite ratings. In healthy individuals, exogenously administered PYY(3-36) suppresses ghrelin (as well as hunger and food intake) [63,64], suggesting a disruption of this interaction in AN. While absolute levels of PYY were higher across the postprandial time-points in patients compared with HCs, consistent with previous reports [15], we found no difference in the magnitude of the PYY response to the meal, suggesting that this was largely accounted for by the elevated baseline levels. This is in contrast to a previous study reporting increased postprandial PYY responses [65], however the inclusion of purging patients in that study may account for these discrepancies, with a recent study reporting elevated postprandial PYY in purging disorder [66]. It is possible that elevated PYY was due to changes in the activity of dipeptidyl peptidase-IV, an enzyme that metabolises PYY, since its serum activity has been reported to be reduced in patients with AN [67], although results are inconsistent [68,69].

In line with previous studies [18,19], we found no difference in baseline or postprandial CCK concentrations during starvation or with refeeding. We also found no difference in baseline or postprandial PP at baseline or with refeeding. Two studies reported elevated baseline and postprandial PP in AN, but had much smaller sample sizes [16,17] and studied medically stable outpatients [16]. Moreover, meal responses were difficult to interpret due to the long duration (up to 50 min) of meal ingestion and the potential cephalic effect on PP release [17]. Our data indicate that CCK and PP are either not involved in mediating disturbances in appetite regulation in AN, or, given that circulating levels are unchanged, patients with AN may be hypersensitive to the anorectic actions of CCK and PP, an effect reduced following refeeding. In support, hypersensitivity to CCK has been reported in patients with functional dyspepsia and in healthy older people with anorexia of ageing [70,71].

Patients with AN exhibited greater GI symptoms, and higher baseline and postprandial bloating compared with HCs at Wk0, which remained unchanged following two weeks of refeeding. In contrast, the perception of fullness was reduced at Wk2. These observations suggest that in AN starvation compromises the patient’s ability to discriminate between feelings of fullness and bloating, and that this capacity is, at least partially, restored by refeeding. We have reported that these patients have markedly slower gastric emptying compared with controls at Wk0 [21], thus, gastric distension was likely exaggerated. In healthy individuals, postprandial fullness is related directly to distension of the distal stomach [8], and patients with AN have greater antral distention than controls, which improves over time during nutritional rehabilitation [3]. We have, however, reported that gastric emptying, assessed using a breath test, was no longer significantly different from controls after two weeks of refeeding [23]. Other studies found no correlation between the improvement in bloating and other GI symptoms with gastric emptying (as measured by antral area) with long-term rehabilitation [3]. Thus, persistent bloating and GI symptoms may contribute to the ongoing difficulties many patients have with eating after weight restoration, potentially contributing to their risk of relapse.

In agreement with previous research [34], patients with AN had substantially higher baseline and postprandial state anxiety than HCs. Following short-term refeeding, baseline anxiety was reduced, while, in contrast, postprandial anxiety was not. Potentially the latter was due to the continued experience of postprandial bloating; in clinical practice the perception of bloating frequently triggers fears of weight gain. The improvement in baseline state anxiety was consistent with previous reports that refeeding and the restoration of body weight results in significant improvement in eating psychopathology, mood and anxiety symptoms [72]. Pre-meal anxiety has been associated with lower caloric intake in patients with AN [33], while improvements in pre-meal anxiety following an exposure and response prevention treatment have been linked to greater food intake [73], thus, targeting pre-meal anxiety may have implications for feeding behaviour. In addition, persistent postprandial anxiety may compromise the recovery process, and potentially present a target for management. In this context, treatment specifically targeting a reduction in postprandial anxiety using relaxation techniques has been reported to diminish postprandial bloating/fullness [74], which may assist with longer-term recovery.

Some study limitations should be recognised. The immediate refeeding required in medically unstable patients with AN may have changed GI hormone responses before studies could be conducted. Patients were only fasted for four hours prior to the study to minimise medical risk, thus, a potential effect of the breakfast consumed on baseline hormone concentrations cannot be excluded. Although calorically identical intakes for each patient over the refeeding period could not be guaranteed, standardised refeeding rates ensured maximal nutrient intake similarity between patients. Because of the relatively small sample size, we were not able to perform statistical analyses to evaluate whether GI hormones or anxiety levels would predict GI symptoms. Follow-up after weight restoration (e.g., up to 12 months) would also be important to determine whether GI hormones, appetite, GI symptoms and anxiety return to healthy levels in the longer-term, or whether some changes, possibly resulting from malnutrition, persist beyond weight restoration.

## 5. Conclusions

Taken together our results indicate that two weeks of refeeding in patients with AN improves their ability to sense hunger and fullness in a manner more similar to HCs, and this may be mediated by changes in GI nutrient sensitivity, while GI symptoms, particularly bloating, and postprandial anxiety persisted. Further studies are required to examine the long-term effects of refeeding, and the impact of these changes in appetite and GI function on treatment success. Furthermore, future studies may examine whether treatment success could be improved by combining refeeding with more targeted treatments aimed at reducing GI symptoms and postprandial anxiety.

## Figures and Tables

**Table 1:** Appetite perceptions, GI symptoms and state anxiety at baseline ^1^.

	HCs	AN Patients		
		Wk0	Wk1	Wk2
Hunger (mm)	56(36–68)	14(5–24) *	35(3–51)	48(2–66)
Desire to eat (mm)	58(43–74)	20(6–59) *	29(2–52)	47(12–65)
Prospective consumption (mm)	60(48–67)	32(21–49) *	39(25–56)	47(31–62)
Fullness (mm)	18(0–48)	65(36–85) *	48(12–73)	39(13–62)
GI symptom score (points)	0(0–2)	7(5–13) *	3(2–8) ^#^	3(0–8) *^#^
Nausea (mm)	3(0–14)	9(3–44)	11(0–35)	2(0–49)
Bloating (mm)	1(0–6)	32(11–73) *	41(11–64)	24(3–49) *
State anxiety (mm)	4(2–13)	27(12–57) *	21(3–65)	9(3–30)

^1^ Data are medians (25th–75th quartiles); *n* = 22 patients with anorexia nervosa (AN) and *n* = 17 healthy controls (HCs). In the patients with AN, main treatment effects were determined using Friedman tests. Comparisons between patients with AN and HCs at both Wk0 and Wk2 were conducted using Mann-Whitney tests. GI, gastrointestinal. * significantly different from HCs, *p* < 0.05; ^#^ significantly different from Wk0, *p* < 0.05.

**Table 2:** Inverse incremental area under the curves (iAUCs) per minute for hunger, desire to eat and prospective consumption perceptions, and iAUCs per minute for fullness perceptions, GI symptoms and state anxiety ^1^.

	HCs	AN Patients		
		Wk0	Wk1	Wk2
Hunger (iAUC, mm)	23(9–40)	10(2–20) *	22(1–39)	26(1–43)
Desire to eat (iAUC, mm)	16(3–32)	10(3–28)	19(1–27)	29(8–35)
Prospective consumption (iAUC, mm)	12(8–21)	16(6–25)	17(3–33)	21(13–38)
Fullness (iAUC, mm)	24(10–42)	16(1–30)	22(10–31)	25(12–40)
Nausea (iAUC, mm)	0(0–2)	0(0–5)	1(0–2)	1(0–4)
Bloating (iAUC, mm)	0(0–2)	6(0–21) *	7(5–19)	12(2–21) *
State anxiety (iAUC, mm)	0(0–0)	2(0–23) *	1(0–7)	3(0–6) *

^1^ Data are medians (25th–75th quartiles); *n* = 22 anorexia nervosa (AN) patients and *n* = 17 healthy controls (HCs). In patients with AN, main treatment effects were determined using Friedman tests. Comparisons between patients with AN at both Wk0 and Wk2 and with HCs were conducted using Mann-Whitney tests. GI, gastrointestinal. * Significantly different from HCs, *p* < 0.05.

**Table 3:** Plasma hormone concentrations at baseline ^1^.

	HCs	AN Patients		
		Wk0	Wk1	Wk2
Acyl ghrelin (pg/mL)	110 ± 11	237 ± 37 *	270 ± 36	261 ± 26 *
CCK ^2^ (pmol/L)	1.2 ± 0.2	1.6 ± 0.2	1.4 ± 0.1	1.8 ± 0.2
Total PYY (pg/mL)	64 ± 6.7	91 ± 8.7 *	84 ± 6.2	80 ± 5.8
PP (pg/mL)	73 ± 42	111 ± 28	106 ± 28	113 ± 28

^1^ Data are means ± SEMs; *n* = 22 patients with anorexia nervosa (AN) and *n* = 17 healthy controls (HCs). In the patients with AN, main treatment effects were determined using one-factor repeated-measures ANOVA with treatment as a within-subjects factor. Comparisons between HCs and patients with AN at both Wk0 and Wk2 were conducted using independent-samples t-tests. CCK, cholecystokinin; PYY, peptide tyrosine tyrosine; PP, pancreatic polypeptide. * significantly different from HCs, *p* < 0.05. ^2^ *n* = 16 HCs and *n* = 19 patients with AN due to missing data.

**Table 4:** Inverse incremental area under the curves (iAUCs) per minute for acyl ghrelin, and iAUCs per minute for CCK, total PYY and PP ^1^.

	HCs	AN Patients		
		Wk0	Wk1	Wk2
Acyl ghrelin (inverse iAUC, pg/mL)	39 ± 6	101 ± 17 *	119 ± 19	112 ± 15 *
CCK ^2^ (iAUC, pmol/L)	1.3 ± 0.2	1.0 ± 0.2	1.5 ± 0.2	1.1 ± 0.2
Total PYY (iAUC, pg/mL)	19 ± 4	17 ± 3	16 ± 3	21 ± 5
PP (iAUC, pg/mL)	51 ± 10	58 ± 13	55 ± 8	54 ± 8

^1^ Data are means ± SEMs; *n* = 22 patients with anorexia nervosa (AN) and n = 17 healthy controls (HCs). In the AN group, main treatment effects were determined using 1-factor repeated-measures ANOVA with treatment as a within-subjects factor. Comparisons between patients with AN and HCs at both Wk0 and Wk2 were conducted using independent-samples t-tests. CCK, cholecystokinin; PYY, peptide tyrosine tyrosine; PP, pancreatic polypeptide. * significantly different from HCs, *p* < 0.05. ^2^ *n* = 16 HCs and *n* = 19 patients with AN due to missing data.
